# Precarious Aging: A Working Definition

**DOI:** 10.1093/geroni/igab046.3633

**Published:** 2021-12-17

**Authors:** Amanda Grenier, Christopher Phillipson, Grace Martin, Abiraa Karalasingam, Karen Kobayashi, Patrik Marier, Debbie Laliberte-Rudman

**Affiliations:** 1 University of Toronto and Baycrest Hospital, Toronto, Ontario, Canada; 2 The University of Manchester, Manchester, England, United Kingdom; 3 University of Toronto, Toronto, Ontario, Canada; 4 University of Victoria, Victoria, British Columbia, Canada; 5 Concordia University, Montreal, Quebec, Canada; 6 Western University, London, Ontario, Canada

## Abstract

Until recently, studies of precarity have overlooked aging and late life. This poster presents a snapshot of conceptual work in progress on a Canadian Social Sciences and Humanities Research Council (SSHRC) Insight Grant on precarity and aging. The poster outlines existing definitions and theoretical perspectives, key results, a current evolving conceptual model, and a working definition of Precarious Aging. It situates existing knowledge and definitions of precarity, highlights crucial intersectional locations of gender, im/migration and (dis)ability, and clarifies the concept of precarity in later life. Results at this point in the study are based on conceptual reviews, reviews of literature on precarity and aging, and the consideration of allied concepts. In conclusion, the concept of precarity offers a promising lens to guide research in the field of social and critical gerontology, providing a foundation for an enhanced understanding of the lives and realities of older people with regards to aging, disadvantage, and inequality.

